# Kisspeptin and LH pulsatility in patients with functional hypothalamic amenorrhea

**DOI:** 10.1007/s12020-020-02481-4

**Published:** 2020-09-11

**Authors:** Agnieszka Podfigurna, Marzena Maciejewska-Jeske, Blazej Meczekalski, Alessandro D. Genazzani

**Affiliations:** 1 Department of Gynecological Endocrinology, Poznan University of Medical Sciences, Poznan, Poland; 2 Department of Obstetrics and Gynecology, Gynecological Endocrinology Center, University of Modena and Reggio Emilia, Modena, Italy

**Keywords:** Kisspeptin, LH, Pulses, Functional hypothalamic amenorrhea

## Abstract

**Purpose:**

Functional hypothalamic amenorrhea (FHA) occurs in response to exaggerated stressors with or without body weight loss. Various hormones, neurotransmitters, and neuromodulators are involved in the control of GnRH and kisspeptin is one of them. Our study aimed to evaluate the putative temporal coupling between kisspeptin and GnRH-induced LH pulsatile secretion.

**Methods:**

In total, 71 patients with FHA were selected for this study. All patients undergo to a pulsatility study for LH and kisspeptin evaluation (120 min, sampling every 10 min), and to an endocrine evaluation for prolactin (PRL), estradiol (E2), androstenedione (A), 17-hydroxy-progesterone (17OHP), TSH, fT3, fT4, insulin, cortisol and testosterone (T), glucose, total cholesterol, triglycerides.

**Results:**

Our data demonstrated kisspeptin and LH pulsatile secretions and that both hormones are co-secreted and temporally coupled at time 0 (*p* < 0.05). When patients were subdivided in hypo-LH (≤3 mIU/ml, *n* = 58) and normo-LH (>3 mIU/ml, *n* = 13), more insights were observed on the specific correlations of metabolic and hormone profiles with pulsatility indexes of LH and kisspeptin.

**Conclusions:**

Our study demonstrated the presence of a distinct kisspeptin episodic secretion in patients with FHA, and showed the temporally coupling of kisspeptin with LH secretory episodes thus supporting that though in amenorrhea, the reproductive axis is still relying on kisspeptin to drive GnRH discharge. In addition, correlations among hormonal data sustain the hypothesis that stress-induced compensatory events are the main direct and indirect promoters of the reproductive blockade in patients affected by FHA.

## Introduction

Secondary amenorrhea is a quite frequent clinical occurrence and when no endocrine or systemic factors are recognized as casual factors, it is usually identified as a hypothalamic blockade. Typically, such hypothalamic dysfunction occurs in all women during fertile life, with no difference between adolescence as well as during adult life [1, 2] and it is considered as functional hypothalamic amenorrhea (FHA). FHA occurs in response to exaggerated metabolic, physical, or psychological stress (after severe dieting, heavy training, intense emotional events, or a combination of them) with or without body weight loss [3–6].

These physical, psychological, and metabolic stressors negatively affect gonadotropin-releasing hormone (GnRH) release and the reproductive axis, activating or inhibiting hypothalamic, and/or extra-hypothalamic areas in brain through neurotransmitters and neuropeptides acting centrally [7]. Various hormones, neurotransmitters, and neuromodulators are involved in the control of GnRH such as prolactin (PRL), cortisol, opioids, noradrenaline, and dopamine [4]. Such negative hypothalamic response is nothing else than a defensive system [7, 8]. In primate females, and in human females in particular, an adaptive mechanism during stress is represented by the reduction of reproductive axis activity, blocking a function which is not essential to survive. Some intermediate steps such as poly- or oligo-menorrhea can anticipate the occurrence of the amenorrheic condition, which is the last and worst stage of this clinical adaptive response to stress [8].

Whatever functional impairment occurs so that to block and blunt gonadotropin release from pituitary, has to do with an impairment of the endogenous GnRH release from hypothalamus [9] with a change in its pulsatile pattern from its classical one characterized by specific frequency and amplitude [10]. GnRH is of cardinal significance for reproductive functions [11] and it determines the proper pulsatile release of follicle stimulating hormone (FSH) and luteinizing hormone (LH) secretion as well as the normal ovarian function. FHA characterized due to abnormal GnRH secretion and related to functional impairments [12] has always to do with three types of stressors: weight loss, psychological stress and exaggerated training/sport [13].

Among the many neuropeptides and neurohormones driving and modulating hypothalamic neurons, kisspeptin is regarded as the potential main player in the physiological and pathological GnRH regulation. Kisspeptin was identified as a peptide encoded by KISS1 gene in 1996 [14]. In fact, the main site of kisspeptin secretion is hypothalamus and more precisely the arcuate nucleus and preoptic region. It plays an essential role in regulation of GnRH secretion.

Kisspeptin is responsible for direct positive influence on GnRH synthesis and release [15] as demonstrated recently by Meczekalski et al. [16]. Indeed healthy eumenorrheic women were demonstrated to have a specific temporal coupling between kisspeptin and LH secretory pulses (occurring in the same time). In addition, such relevant physiological temporal linkage was also reported to be impaired in a classic gynecological–endocrinological disease that is polycystic ovary syndrome (PCOS) [17].

Since recently abnormal and reduced kisspeptin plasma concentration were demonstrated in patients suffering for FHA [18], we evaluated if any temporal coupling might be demonstrated between kisspeptin and LH secretory episodes in a group of patients with FHA.

## Material and methods

### Patients and study design

Seventy one amenorrheic patients (*n* = 71), mean age of 21 ± 1.0 (mean ± SEM) years were selected for this study, after giving their informed consent, among those referring to the Center for Gynecological Endocrinology, University of Poznan, Poland, for a secondary amenorrhea.

Patients were enrolled excluding any ongoing diseases and on the basis of the following criteria: (1) the presence of amenorrhea in the last 6 months, (2) no metabolic diseases, (3) body weight stable in the last 6 months and within the normal ranges for age and height and with a body mass index (BMI) not below 19 kg/m^2^, (4) history of emotionally stressful events preceding the onset of amenorrhea, such as problems within the family, at school, at work or of psycho-social stress; psychiatric diseases were excluded using DSM-V criteria [19], (5) no intense training for agonistic purposes, (6) exclusion of adrenal, thyroid or PRL diseases, on the basis of hormonal profiles. PCOS, independently from the ultrasound investigation, was excluded on the basis of the combined presence of low androgen plasma levels and of the lack of clinical signs of androgenization, as according to Rotterdam Consensus Conference [20].

All patients were invited not to change their life-style and to undergo an endocrine evaluation as follows: LH, FSH and kisspeptin pulsatility profiles (120 min, sampling every 10 min), PRL, estradiol (E2), 17-hydroxy-progesterone (17OHP), TSH (thyroid-stimulating hormone), fT3, fT4, insulin, cortisol and testosterone (T), glucose, total cholesterol, triglyceride.

Vaginal US was performed before and after the treatment to evaluate the changes of the thickness of the endometrium.

Each patient included in the study had blood drawn from a vein in the arm (5 ml) to a dry, plastic tube in the morning (between 6.00 and 10.00 a.m.) and the patients were fasting. After collection and clot formation, the blood was centrifuged at acceleration of 1500 g for 10 min to obtain serum for baseline endocrine evaluation. In addition, an intravenous heparin lock was inserted in an antecubital vein at 7 a.m., with the start of the sampling collection at 8 a.m. to perform a pulsatility. In total, 12 blood samples were collected between 8.00 and 10.00 a.m. in the amount of about 1 ml from each patient at 10-min intervals during the following 2 h, for a total of 13 blood samples, to determine kisspeptin and gonadotropins plasma concentrations and to evaluate the putative presence of a spontaneous pulsatile release of these hormones.

Similarly to previous studies [21, 22], patients were subdivided in two groups according to LH plasma concentrations: those with LH ≤ 3 mIU/ml (*n* = 58) and those with LH > 3 mIU/ml (*n* = 13).

The study design was approved by the Ethical Committee of the University of Poznan, Poland.

### Assay

Kisspeptin was measured with the use of enzyme-linked immunosorbent assay (ELISA) and a Kiss-1 (112–121) Amide/Kisspeptin-10/Metastatin (45–54) Amide (Human) EIA Kit 1 (Phoenix Pharmaceuticals). This ELISA kit provides 100% cross-reactivity with longer kisspeptin 1–54 forms, so it detects both forms of active kisspeptin, as previously described [16]. Kisspeptin samples from each subject were analyzed in the same assay. The interassay coefficient of variation (CV) for all hormonal assays was <9% at the concentrations measured. Intra-assay CV for LH was 6.8% with a sensitivity of 0.8 ng/mL, as previously described [16].

Serum FSH, LH, PRL, E2, T, cortisol, DHEAS, TSH, fT4, and insulin serum concentrations were determined by means of electrochemoluminescence immunoassay (Roche Diagnostics). Concentrations of various serum hormones were measured with the use of a Cobas E601 analyzer (Roche Diagnostics). Intra and interassay %CV for all these assays were 4% and 7%, respectively.

Total CRP, cholesterol, glucose and triglycerides were assayed by standard assay system of the Centralized Lab of Poznan University Hospital, Poland.

### Pulse detection and degree of concordance

#### Pulse detection

Time series of LH and kisspeptin were first evaluated separately to calculate the random measurement error on the duplicates of each time series using the program Predetec, a specific program of Detect software. Predetec calculates the mean (*X*) and variance (*s*^2^_x_) for each set of replicates in the time series. It plots *s*^2^_x_ or *s*_x_ versus *X*, or log(*s*^2^_x_) versus log(*X*) and then fits five different models to this relationship, selecting the best model in terms of the smallest root-mean-square error for *s*_x_, as previously described [23]. The program is subject to the constraint that the predicted *s*_x_ cannot be negative for the observable range of the data. Finally, the program provides coefficients for the variance model to be used in the Detect program for pulse detection analysis [23, 24]. Detect identifies the secretory episodes on each time series with a *p* value equal to 0.01 (1%) for the nominal false-positive rate, as previously described [23, 24].

#### Degree of concordance

The presence of significant concomitance between the secretory events of LH and kisspeptin was assessed by computing the specific concordance (SC) index, interposing various lags between each time series couplet under analysis [25]. The time series of each hormone (A and B) was converted into a “quantized” time series, where only the first sample (onset) of each detected peak was taken to represent the occurrence of that peak [25]. The quantized data series was then matched, and quantized values for hormone A were compared to the corresponding values for hormone B. The presence or absence of an event (onset) in either or both series was counted and the SC index was then computed. A spectrum of SC values was constructed by sliding the two series, interposing integral multiples of the sampling interval as the lag. The “0” point represents the first event (peak) from which the two temporal series are matched and then slided interposing then various lags of time (three time lags before and three time lag after, that is 30 min before or after). The presence of a positive lag time indicates that an event in A preceded the secretion event in B [25].

SC spectra were evaluated for each subject and the mean SCs over each group of patients were calculated at each lag time, obtaining a mean SC spectrum. The location of the maximum of the mean SCs for each group of women was also noted. Monte Carlo simulations were then performed to study the frequency distribution of the SC index under the null hypothesis of random concordance, as previously described. For each of the 2 pairs of clinical data for each subject, 500 simulated pairs of simulated series were generated and frequency distributions of SC obtained. An SC value above the 95% percentile of the frequency distribution generated for that individual or group of subjects resulted in rejection of the null hypothesis at the *p* < 0.05 confidence level [25].

#### Statistical analysis

Data are expressed as mean ± SEM. We tested data for significant differences between groups, after analysis of variance (one-way ANOVA), using Student’s *t* test for paired data. Pearson’s index was computed to evaluate correlation coefficients between groups.

Correlation (Pearson) index was computed to evaluate the presence of any correlation between variables under consideration. A *p* level < 0.05 was considered significant.

## Results

### Hormonal parameter

Table 1 (upper panel) summarizes the hormonal profile of the whole set of patients under study (*n* = 71). When patients were subdivided according to baseline LH plasma levels, hypo-LH (Table 1, middle panel) subjects differ from normo-LH subjects for the lower gonadotropin, testosterone, and HDL plasma levels (Table 1, lower panel). On the contrary, BMI was similar in both groups of patients.

**Table 1:** Hormonal and metabolic parameters of subjects with FHA under study

	LH mIU/mL	FSH mIU/mL	E2 pg/mL	T ng/mL	PRL ng/mL	TSH µIU/mL	fT4 ng/dL	Cortisol nmol/L	DHEAS µmol/L	Insulin µU/mL	Glucose mg/dL	Total Cholest mg/dL	LDL mg/dL	HDL mg/dL	Triglic mg/dL	CRP mg/L	BMI (kg/m²)
All patients (*n* = 71)	1.7 ± 0.1	4.3 ± 0.2	16.4 ± 1.5	0.2 ± 0.01	8.4 ± 0.5	1.7 ± 0.08	1.2 ± 0.05	458.6 ± 16.6	7.2 ± 0.2	5.9 ± 0.3	84.6 ± 1.0	191.7 ± 4.1	100.8 ± 4.3	79.7 ± 2.6	73.5 ± 3.3	1.1 ± 0.4	19.8 ± 0.2
Hypo-LH (*n* = 58)	1.2 ± 0.1	4.0 ± 0.2	16.8 ± 1.7	0.2 ± 0.01	8.0 ± 0.6	1.6 ± 0.09	1.2 ± 0.07	466.6 ± 18.7	7.1 ± 0.2	6.1 ± 0.3	84.6 ± 1.2	191.3 ± 4.4	103.6 ± 4.8	76.4 ± 2.5	73.2 ± 3.6	1.2 ± 0.5	19.7 ± 0.2
	****	***		*										**			
Normo-LH (*n* = 13)	4.1 ± 0.1	5.6 ± 0.5	15.0 ± 3.7	0.3 ± 0.04	10.0 ± 1.2	1.9 ± 0.2	1.2 ± 0.05	402.5 ± 33.4	7.5 ± 0.6	5.0 ± 0.5	83.7 ± 0.2	197 ± 11.1	89.0 ± 8.4	97.2 ± 7.8	75.0 ± 8.3	0.4 ± 0.1	19.8 ± 0.2

****p < 0.000001; ***p < 0.001; **p < 0.005; *p < 0.01 vs normo-LH patients

### Pulse analysis and SC (specific concordance) index determination

When LH and kisspeptin pulsatility profiles were analyzed using Detect algorithm, specific secretory episodic release was observed (Table 2) though in some subjects either LH or kisspeptin resulted not to have significant secretory peaks. Over 71 patients, LH and kisspeptin secretory episodes were significantly evidenciated by Detect algorithm in 59 patients (*n* = 47 hypo-LH and *n* = 12 normo-LH) and in 65 patients (*n* = 52 hypo-LH and *n* = 13 normo-LH), respectively (Table 2). The lack of significant pulse was due to the low hormonal plasma concentrations of that pulsatile profile and also to the small pulse amplitude of the secretory pulses. When a change of the plasma concentrations constituting the pulsatility profile did not reach the threshold that Detect algorithm set for the pulse analysis, no significant peak is detected. Such logic disclosed that among hypo-LH patients there are some subjects with no detectable peaks in either LH or kisspeptin pulsatilities (6 patients for kisspeptin, 11 patients for LH, Table 2, middle panel). Such problem was almost not occurring (1 subject only for kisspeptin) in the normo-LH patients.

**Table 2:** LH and Kisspeptin pulse parameters

	Integrated plasma concentrations LH mIU/ml Kisspeptin ng/ml	Pulse frequency/2 h
All patients (*n* = 71)		
Kisspeptin (*n* = 65)	1.8 ± 0.1	2.2 ± 0.1
LH (*n* = 59)	1.7 ± 0.1	2.5 ± 0.2
Hypo-LH (*n* = 58)		
Kisspeptin (*n* = 52)	1.7 ± 0.1**	2.2 ± 0.1
LH (*n* = 47)	1.3 ± 0.1***	2.4 ± 0.2
Normo-LH (*n* = 13)		
Kisspeptin (*n* = 13)	2.6 ± 0.3	2.2 ± 0.3
LH (*n* = 12)	3.3 ± 0.4	2.4 ± 0.4

Close to each hormone the number of studied pulsatilities is reported

**p < 0.01; ***p < 0.000001 vs normo-LH patients

In this regards it has to be pointed out that though significant secretory events (i.e., peaks) were not detected in some patients, Detect program, after analysis of variance on the whole set of data of these pulsatilities, demonstrated that data were not random at the 0.05 *p* level [20]. This means that the integrated mean computed over the 13 concentrations points of the pulsatility showed a standard deviation higher than that expected according to the CV of the assay. Though no distinct pulses were detected, the evaluation of this variance model all over the pulsatility demonstrated the presence of concentration changes (i.e., the increase due to the peaks) in kisspeptin or LH pulsatile profiles that are not random variations nor due to an assay variability and thus can be indirectly supposed to be related to the presence of hormonal pulses that, however, being very small in amplitude, do not reach the statistical significance for the Detect algorithm and were not entitled to be labeled as a “peak”.

When subjects were considered according to LH plasma concentrations, hypo-LH patients showed an integrated concentrations (computed as the mean of the whole set of 13 points of the pulsatility) lower than that of normo-LH subjects for both LH and kisspeptin (Table 2, middle and lower panel). On the contrary the pulse frequency over the 2 h of pulsatility study resulted perfectly similar (Table 2).

**Fig. 1:**
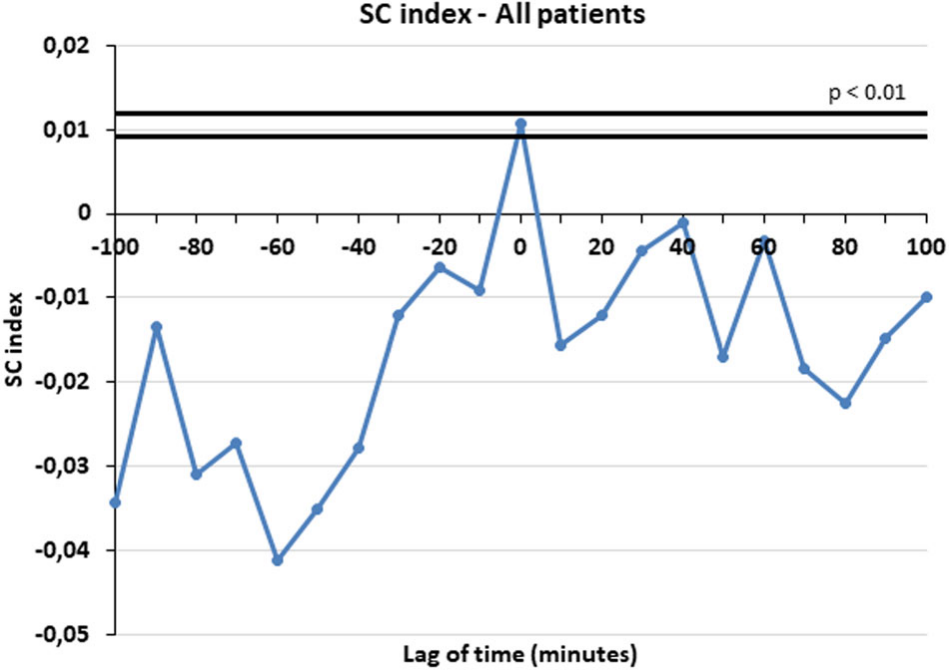
Specific concordance (SC) index at time 0 between LH and kisspeptin in all patients

**Fig. 2:**
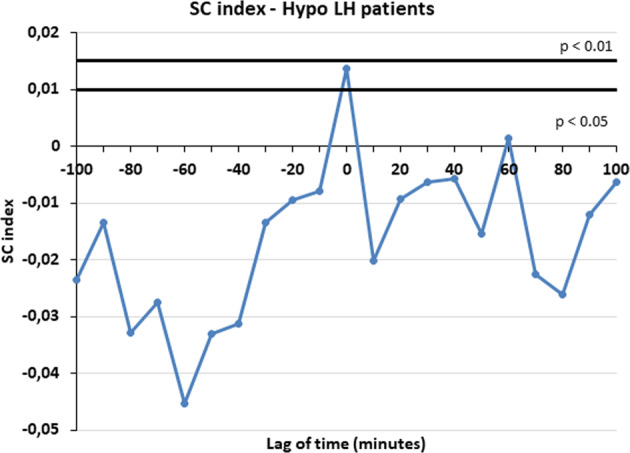
Specific concordance (SC) index at time 0 between LH and kisspeptin in hypo-LH patients

The concordance between LH and kisspeptin pulses was computed on the pulsatility profiles of the patients that had peaks detected in both time series (i.e., LH and kisspeptin). This analysis gave the consistent prove that the two hormones have a significant SC index at time 0 (*p* < 0.05). This means that kisspeptin and LH are co-secreted exactly in the same moment and the concordance is at time lag 0 (Fig. 1). When such analysis was performed considering LH plasma concentrations, hypo-LH patients showed exactly the same significant concordance at time lag 0 (Fig. 2) (*p* < 0.05). Unfortunately, the normo-LH group was characterized by a low number of subjects and this consistently reduced the statistical power of the data and no significant concordance was observed, though being the SC index higher at time lag 0 than in any other time lag of the observation interval (from −100 to +100 min).

### Pearson index

#### All patients (*n* = 71) (Table 3)

Integrated kisspeptin plasma concentrations were positively correlated with kisspeptin pulse frequency and negatively with cortisol plasma concentrations while were very close to significance with integrated LH plasma concentrations; integrated LH plasma concentrations were significantly correlated with BMI, E2 and insulin plasma concentrations and were negatively correlated with cortisol plasma concentrations; LH pulse frequency was positively correlated with E2 plasma concentrations and kisspeptin pulse frequency and negatively correlated with cortisol plasma concentrations; glucose plasma concentrations were positively correlated with insulin, cholesterol and cortisol plasma concentrations.

**Table 3:** Pearson’s (correlation) index among the various variables on the whole group of patients (*n* = 71)

*p* level	*r*	Variables
*p* < 0.01	0.29161069	Integr Kiss & Kiss pulses
NS	0.13767296	Bmi & integr Kiss
NS	−0.0076849	BMI & Kiss pulses
*p* < 0.05	0.29366933	BMI & integr LH
NS	0.05454914	BMI & LH pulses
*p* < 0.01	0.35487483	E2 & intrgr LH
*p* < 0.05	0.20214838	E2 & LH pulses
NS	−0.00065346	E2 & integr Kiss
NS	0.09287813	E2 & Kiss pulses
NS	0.1045219	E2 & T
*p* < 0.05	0.24632567	Ins & integr LH
NS	−0.16267834	Ins & LH pulses
NS	−0.07302149	Ins & integr Kiss
NS	0.00534334	Ins & Kiss pulses
NS	−0.206753	Ins & DHEAS
*p* < 0.0001	0.48152951	Ins & glucose
*p* < 0.001	0.3150945	Glucose & colest
*p* < 0.05	−0.138118	Cortisol & LH pulses
*p* < 0.05	−0.23279208	Cortisol & integ LH
NS	0.04974289	Cortisol & Kiss pulse
*p* < 0.001	−0.41958448	Cortisol & integr Kiss
*p* < 0.01	0.31101492	Cortisol & glucose
NS	0.14033864	Cortisol & col tot
NS	0.02391467	Cortisol & DHEAS
NS	0.00631198	Integr LH & LH pulses
NS	0.20114874	Integr kiss & integr LH
*p* < 0.05	0.26746294	Kiss pulses & LH pulses

#### Hypo-LH patients (*n* = 58) (Table 4)

Integrated kisspeptin plasma concentrations were positively correlated with kisspeptin pulse frequency and negatively with cortisol plasma concentrations; integrated LH plasma concentrations were significantly correlated with BMI, E2, and insulin plasma concentrations; LH pulse frequency was positively correlated with E2 plasma concentrations; glucose plasma concentrations were positively correlated with insulin, cholesterol and cortisol plasma concentrations.

**Table 4:** Pearson’s (correlation) index among the various variables on hypo-LH patients (*n* = 58)

*p* level	*r*	Variables
*p* < 0.01	0.32457521	Integr Kiss & Kiss pulses
NS	0.11127047	BMI & integr Kiss
NS	−0.07569535	BMI & Kiss pulses
*p* < 0.05	0.26237856	BMI & integr LH
NS	−0.01836273	BMI & LH pulses
*p* < 0.001	0.49777592	E2 & intrgr LH
*p* < 0.05	0.31492295	E2 & LH pulses
NS	0.01805854	E2 & integr Kiss
NS	0.05878813	E2 & Kiss pulse freq.
NS	0.0787185	E2 & T
*p* < 0.001	0.48546361	Ins & integr LH
NS	−0.17719212	Ins & LH pulse freq.
NS	−0.01931863	Ins & integr Kiss
NS	0.05748145	Ins & Kiss pulse freq.
NS	−0.12736682	Ins & DHEAS
*p* < 0.001	0.48025007	Ins & glucose
*p* < 0.01	0.37533613	Glucose & colest
NS	−0.09587902	Cortisol & LH pulse freq.
NS	−0.16802432	Cortisol & integ LH
NS	0.18520289	Cortisol & Kiss pulse freq.
*p* < 0.01	−0.36149893	Cortisol & integr Kiss
*p* < 0.05	0.3022964	Cortisol & glucose
NS	0.18400018	Cortisol & col tot
NS	0.04570806	Cortisol & DHEAS
NS	−0.05112006	Integr LH & LH pulse freq.
NS	0.00054127	Integr kiss & interg LH
NS	0.21173808	Kiss pulse & LH pulse freq.

#### Normo-LH patients (*n* = 13) (Table 5)

This subgroup of patients with hypothalamic amenorrhea showed some though not all of the correlations observed for hypo-LH subjects. Due to the low number of subjects of this group there was a low statistical power and this did not permit to disclose more insights in regards to the correlations among the various variables under study. In fact, integrated LH concentrations were positively correlated with BMI and E2 concentrations; insulin plasma concentrations were positively correlated with glucose and negatively with DHEAS plasma concentrations; integrated kisspeptin plasma concentrations were negatively correlated with cortisol plasma levels.

**Table 5:** Pearson’s (correlation) index among the various variables on the subgroup of normo-LH patients (*n* = 13)

*p* level	*r*	Variables
NS	0.19989739	Integr Kiss & Kiss pulses
NS	0.15120382	BMI & integr Kiss
NS	0.19802503	BMI & Kiss pulses
*p* < 0.05	0.62347405	BMI & integr LH
NS	0.47153624	BMI & LH pulses
*p* < 0.05	0.581676	E2 & intrgr LH
NS	−0.27654956	E2 & LH pulses
NS	−0.15278229	E2 & integr Kiss
NS	0.28962528	E2 & Kiss pulses
NS	0.29606054	E2 & T
NS	0.11468259	Ins & integr LH
NS	−0.08435475	Ins & LH pulses
NS	−0.06633135	Ins & integr Kiss
NS	−0.25477883	Ins & Kiss pulses
*p* < 0.001	−0.7578863	Ins & DHEAS
*p* < 0.05	0.5680017	Ins & glucose
NS	−0.0473531	Glucose & colest
NS	−0.20025325	Cortisol & LH pulses
NS	−0.19827253	Cortisol & integ LH
NS	−0.50676674	Cortisol & Kiss puls
*p* < 0.05	−0.61852654	Cortisol & integr Kiss
NS	0.25886539	Cortisol & glucose
NS	−0.13964439	Cortisol & col tot
NS	0.19459678	Cortisol & DHEAS
NS	0.26582677	Integr LH & LH pulses
NS	0.07863105	Integr kiss & interg LH
NS	0.45894103	Kiss pulses & LH pulses

## Discussion

This study assessed the presence of a distinct spontaneous episodic release of kisspeptin and its temporal coupling with LH secretory pulses in patients with FHA.

Jayasena et al. [26] published that subcutaneous injection of kisspeptin was able to induce gonadotropin secretion as well as estradiol rise in women suffering for FHA thus supporting the hypothesis that kisspeptin stimulates gonadotropins release and that its one of the neurohormones driving reproduction [17]. In addition, in FHA, the lack of an adequate kisspeptin secretion might be at the basis of the reproductive blockade [17].

Interestingly, chronic kisspeptin administration resulted in desensitization to its effects on gonadotropin release. Conversely, when kisspeptin was administered in FHA patients twice weekly for 8 weeks, it was reported the significant increase of all reproductive hormones plasma levels after 8 weeks of treatment with no side effects [17].

Our data indirectly support such reports [17, 26] since in our study the presence of the reproductive blockade in FHA parallel a significant impairment of both kisspeptin and LH secretion. In fact, the subdivision of the patients according to the LH plasma levels clearly disclosed that in patients with very low LH plasma levels there were very low kisspeptin concentrations.

Pulse analysis assessed not only the presence of a distinct LH episodic secretion in the FHA patients but also demonstrated for the first time that there is a significant temporal relationship between kisspeptin and LH pulsatile secretions. Our present data confirm the presence of LH pulsatile release in hypogonadal FHA patients similarly to what previously reported [21, 22] and disclosed the presence of a distinct episodic release also for kisspeptin as previously reported in healthy women as well as in PCOS patients, even though with specific peculiarities [16, 27]. Our study clearly demonstrated that the kisspeptin pulsatile secretion is present independently from the kisspeptin plasma concentrations and that LH plasma levels parallels kisspeptin plasma levels, being LH levels low exactly when kisspeptin levels are low. The real difference between the hypo-LH and normo-LH groups was in the LH and kisspeptin integrated plasma concentrations. Such difference is easily understandable since it was due to the lower pulse amplitude of both LH and kisspeptin.

When the temporal coupling between LH and kisspeptin pulses was investigated, a temporal coupling was demonstrated at time “0”. This indicates that at each kisspeptin secretory burst, an immediate LH secretory pulse occurs, with no lag of time, similarly to eumenorrheic women and PCOS patients (16, 27). Our data together with previous reports are clear demonstration that kisspeptin is at a high probability the biological trigger of GnRH discharge and of simultaneous LH pulsatile release, even though it cannot be excluded that also leptin might play a role on such regulation. In fact, leptin signal transduction pathway includes kisspeptin. Unfortunately in our present study leptin concentrations were not determined but it remains that kisspeptin role seems to be a key factor to get an appropriate GnRH secretion from hypothalamus.

Our study disclosed also the finding of a potential connection between the hormonal and the metabolic profiles in patients with FHA. Our data disclosed a close relationship between the reproductive axis and the metabolic homeostasis of the patients with FHA as for the presence of a positive correlation between LH levels and BMI values, estradiol and insulin concentrations in blood serum [28].

Moreover, the evidence of the connection between the reproductive axis and the hypothalamic–pituitary–adrenal (HPA) axis is indicated by the negative correlation between the concentration and pulse frequency of kisspeptin and serum cortisol plasma levels. In our FHA patients with low integrated kisspeptin levels but normal pulse frequency (similar to what reported in healthy subjects) [16], increased levels of cortisol were observed. The higher is cortisolemia the lower is kisspeptin plasma level. In fact, the HPA axis is known as our central stress response system. In response to stressors, ACTH-induced cortisol is released, even in higher amounts, thus negatively acting on the hypothalamus–pituitary–ovarian axis [29] and thus participating to the reproductive blockade of FHA.

In patients with FHA a strong relationship between metabolism, stress, and reproduction exist. The best evidence of this, are the numerous positive and negative correlations found between BMI, serum concentrations of cortisol, insulin, glucose, LH, and kisspeptin. Depending on the value, both LH and kisspeptin concentrations closely correlate with specific parameters such as cortisol. This relationship undoubtedly confirms the tight relationship and the negative role of the hyperactivation of HPA axis on the reproductive axis [30].

Of interest from the present study, is the relationship between serum insulin and DHEAS concentrations. Patients with FHA, with normal serum LH levels (above 3 mIU/ml), showed a negative correlation of serum insulin with DHEAS, which was not observed in patients with low LH levels. Such observation might be because the two groups of patients with FHA represent two distinct moment of the clinical evolution of the same physiopathological condition. Those with normal LH levels are at the first stages of the disruption, relatively affected by the stress-induced adaptive response, while those with low LH levels are probably the group whose adaptive response to stress have already impaired the biological and normal control of the reproduction.

In conclusion, our study clearly supports the evidence of a dynamic pulsatile release of kisspeptin in patients with FHA, reporting that the episodic discharge is temporally coupled with LH thus demonstrating that the reproductive axis, also in FHA, relies on kisspeptin to drive GnRH discharge.
